# DupliPHY-Web: a web server for DupliPHY and DupliPHY-ML

**DOI:** 10.1093/bioinformatics/btu645

**Published:** 2014-10-07

**Authors:** Ryan M. Ames, Simon C. Lovell

**Affiliations:** Faculty of Life Sciences, University of Manchester, Oxford Road, Manchester M13 9PL, UK

## Abstract

**Summary**: Gene duplication and loss are important processes in the evolution of gene families. Moreover, growth of families by duplication and retention is an important mechanism by which organisms gain new functions. Therefore the ability to infer the evolutionary histories of families is an important step in understanding the evolution of function. We have recently developed DupliPHY, a software tool to infer gene family histories using parsimony and maximum likelihood. Here, we present DupliPHY-Web a web server for DupliPHY that implements additional maximum likelihood functionality and provides users an intuitive interface to run DupliPHY.

**Availability and implementation**: DupliPHY-Web is available at www.bioinf.manchester.ac.uk/dupliphy/

**Contact**: ryan.ames@manchester.ac.uk

**Supplementary information**: Supplementary data are available at *Bioinformatics* online.

## 1 INTRODUCTION

Ohno (1970) first proposed that gene or genome duplication is a major mechanism for generating functional innovation and genome evolution. For eukaryotes it has been estimated that 50% of genes are expected to duplicate at least once in time scales of the order of 35–350 million years (Lynch and Conery, 2000). Indeed, up to 30% of the yeast genome may have arisen by duplication events (Rubin *et al.*, 2000). There is also evidence for whole genome duplication (WGD) in yeast (Wolfe and Shields, 1997), which was followed by rapid and extensive gene loss (Kellis *et al.*, 2004). Gene duplication also seems to have been important in the evolution of humans (McLysaght *et al.*, 2002).

Determining the evolution of gene families and identifying the gain and loss events is, therefore, necessary to understanding the processes of gene family evolution. Previously, we have released the software DupliPHY (Ames *et al.*, 2012) that provides weighted parsimony and maximum likelihood methods to accurately infer the evolutionary histories of gene families. The software has been used to identify lineage-specific expansions of gene families in yeast (Ames *et al.*, 2014). DupliPHY works on gene copy number counts to infer gene family evolution and is different from tree reconciliation methods that infer duplication and loss events based on gene and species trees (Larget *et al.*, 2010). The tool requires a file describing the membership of gene families for a range of species and a phylogenetic tree describing the evolutionary relationship between these species.

The DupliPHY programs are written in Java v1.7 and are run from the command line. Here, we introduce DupliPHY-Web, a web interface for the DupliPHY programs that provides users with a visual method of running the programs without any local installation. There is substantial help documentation and a walkthrough for novice users, including advice on the interpretation of the results. Additionally, DupliPHY-ML has been updated with a new model of gene family evolution and to make the program more configurable. Ultimately, DupliPHY-Web will provide a useful tool for analysing gene family evolution.

## 2 IMPLEMENTATION

### 2.1 The web server

**Fig. 1.**
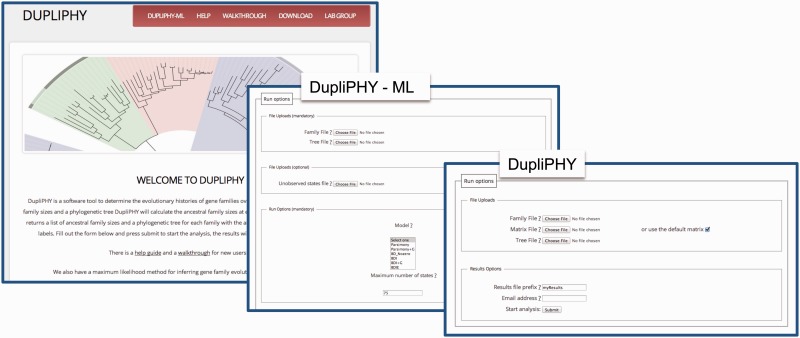
The DupliPHY-Web interface showing the analysis options for both DupliPHY and DupliPHY-ML. The user can upload the relevant data files and start the analysis. The results of the analysis are then emailed to the supplied email address

DupliPHY-Web is a web interface which can be used to run the DupliPHY programs (Fig. 1). The run options for both programs are divided into mandatory and optional parameters. The web interface ensures that all necessary parameters have been selected before the data are submitted to the server. To run either DupliPHY program the user will need a family file and a phylogenetic tree. Descriptions of the file formats as well as instructions of how to generate the underlying data for these files can be found in the help documentation.

Once the user has submitted his/her data, DupliPHY-Web validates the input files to check for errors. The validation checks the format and consistency of all options and files uploaded. If any errors are encountered the user is immediately notified and can then upload the corrected files. As DupliPHY-ML may be slow on large datasets, the DupliPHY-ML analysis on the server has been limited to <10 000 families, no families with >75 members and the analysis must contain <100 species. Once the input files have passed the validation stage, the tool starts the analysis.

The results of the analysis are emailed to the user. After running DupliPHY the user will receive a file containing the ancestral reconstruction for each family, which shows the size of each family at each node of the tree. DupliPHY also returns a tree file with one tree per family. Here, the internal nodes are labelled with the ancestral family sizes and can be viewed using a phylogenetic tree viewer. DupliPHY-ML also returns a file reporting the ancestral sizes for each family. In addition, DupliPHY-ML returns a tree file containing the input phylogenetic tree where the branch lengths represent the average number of inferred events on that branch. The results also include files containing the estimated parameters for the model used, the mean posterior rates for each family and the probabilities for each of the inferred ancestral family sizes. For both programs the walkthrough guide contains descriptions of the results files produced.

### 2.2 Updates to DupliPHY-ML

DupliPHY-ML has been updated to be more configurable, allowing the user to choose a specific optimizer, whether to fix or infer branch lengths, and to choose the method of ancestral reconstruction. Perhaps most notably, DupliPHY-ML now contains a new model of gene family evolution that contains a parameter for the extinction of a family. Here, extinction refers to an event where a family is completely lost from the genome. This family can subsequently only be regained by an innovation event. It is important to consider the extinction of gene families as these events are common, there have been an estimated 12 extinctions per million years in *Drosophila* (Hahn *et al.*, 2007). The new model is the Birth-Death-Innovation-Extinction (BDIE) model, and is defined by the instantaneous rate matrix ***Q***:

(1) $$Q_{i,j}=\{\begin{array}{l} b\text{ if }j-i=1\text{ and }i\neq 0(\text{birth})\\ d\;\text{if}\;i-j=1\text{ and }j>0(\text{death})\\ h\text{ if }i=0\;\text{and}\;j=1(\text{innovation})\\ k\text{ if}\;j=0\;\text{and}\;i=1(\text{extinction})\\ 0\text{ if }|i-j|>1(\text{maximum one event}) \end{array}$$

The BDIE model is more biologically relevant than our previous models (Ames *et al.*, 2012) and aims to describe more processes in the evolution of gene families (Ames *et al.*, 2014). We benchmarked both the BDI and BDIE models on simulated data and found both models can accurately infer ancestral gene family sizes (Supplementary File 1). As the run time of these models varies the BDI model is recommended for large datasets that may result in long run times, whereas the BDIE model should be used in cases where extinction maybe common. Models that encompass even more biological realism, such as segmental or WGD events, will be important for future research.

## 3 CONCLUSIONS

Inferring the evolutionary histories of families is an important step in understanding the evolution of function. DupliPHY-Web provides an intuitive web interface for the DupliPHY programs, enabling users to conveniently run gene family evolution analyses. Users have access to extensive help documentation, and future versions of DupliPHY, including the addition of new models, can be deployed directly to the server.

## Supplementary Material

Supplementary Data
