# Supplementary material for: DupliPHY-Web: a web server for DupliPHY and DupliPHY-ML
Source: Bioinformatics. 2014 Oct 7;31(3):416–7. doi: 10.1093/bioinformatics/btu645 (PMC4308661; doi:10.1093/bioinformatics/btu645)
Supplement: Supplementary Data [file supp_31_3_416__index.html]

DupliPHY-Web: A web server for DupliPHY and DupliPHY-ML. — DupliPHY-Web: a web server for DupliPHY and DupliPHY-ML — DupliPHY-Web: a web server for DupliPHY and DupliPHY-ML — Supplementary Data 

# DupliPHY-Web: a web server for DupliPHY and DupliPHY-ML

## Supplementary Data

files

**Files in this Data Supplement:**

- Supplementary Data - pdf file
