# Supplementary material for: DupliPHY-Web: a web server for DupliPHY and DupliPHY-ML
Source: Bioinformatics. 2014 Oct 7;31(3):416–7. doi: 10.1093/bioinformatics/btu645 (PMC4308661; doi:10.1093/bioinformatics/btu645)
Supplement: Supplementary Data [file supp_btu645_supp_file_1.pdf]

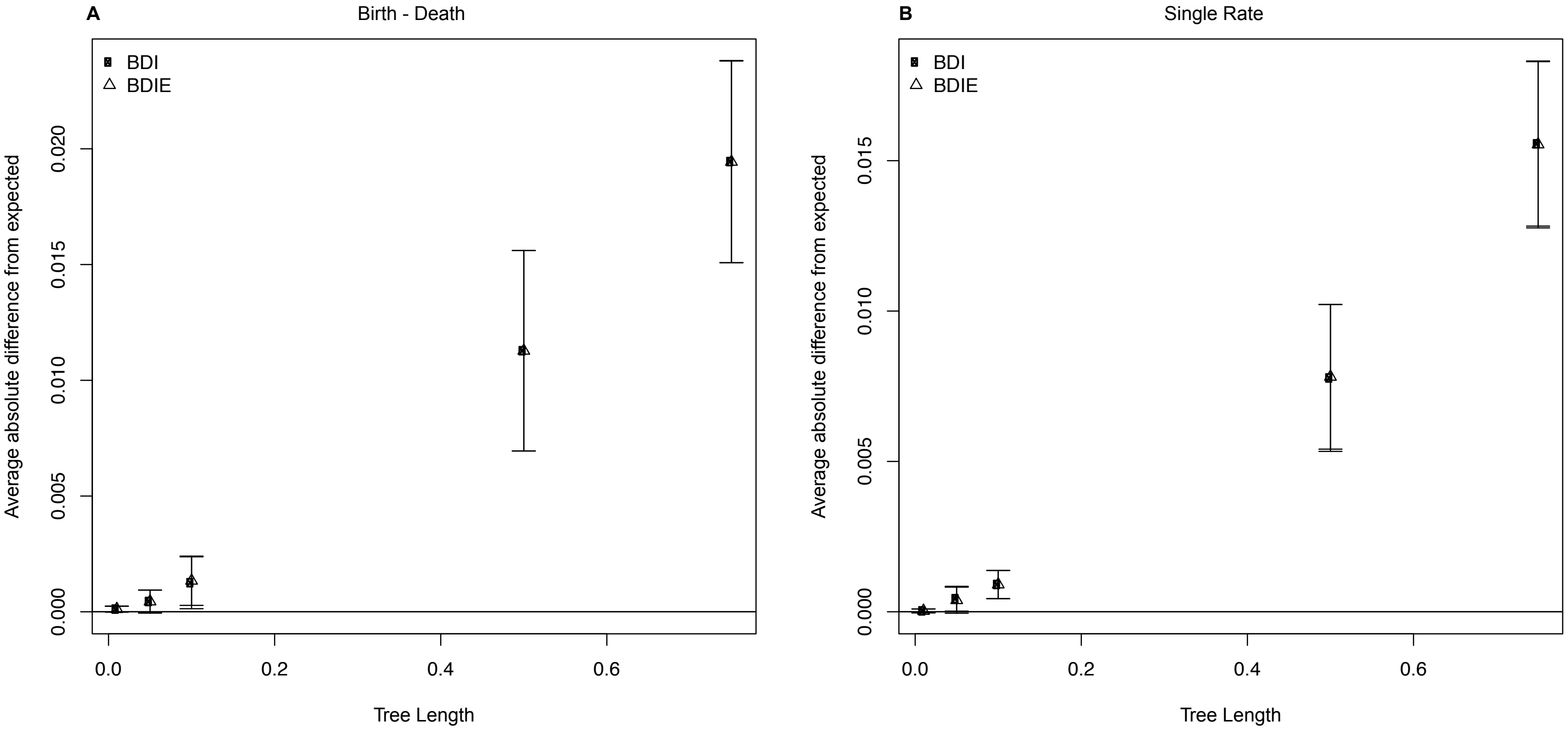

Figure 1: The accuracy of the birth-death-innovation (BDI) and birth-death-innovation-extinction (BDIE) models on determining ancestral gene family sizes. Inferences were made over 5 tree lengths each with 10 repetitions containing 1481 gene families. Expected data were simulated under a birthdeath and single rate models as described in Ames *et al.* 2012. The closed squares and open triangles represent the performance of the BDI and BDIE models respectively. Error bars are SDs. The line at 0 represents the simulated value.
